# Epstein–Barr Virus-Associated Malignancies: Roles of Viral Oncoproteins in Carcinogenesis

**DOI:** 10.3389/fonc.2018.00265

**Published:** 2018-08-02

**Authors:** Ahmed El-Sharkawy, Lobna Al Zaidan, Ahmed Malki

**Affiliations:** ^1^Human Molecular Genetics Laboratory, Institute of Genetics and Biophysics “A. Buzzati-Traverso” (IGB)-CNR, Naples, Italy; ^2^Biomolecular Science Programme, Università Degli Studi Della Campania “Luigi Vanvitelli”, Naples, Italy; ^3^Biomedical Science Department, College of Health Sciences, Qatar University, Doha, Qatar

**Keywords:** Burkitt’s lymphoma, nasopharyngeal carcinoma, B-cells lymphoma, Hodgkin’s lymphoma, non-Hodgkin’s lymphoma, oncoproteins, oncogenes

## Abstract

The Epstein–Barr virus (EBV) is the first herpesvirus identified to be associated with human cancers known to infect the majority of the world population. EBV-associated malignancies are associated with a latent form of infection, and several of the EBV-encoded latent proteins are known to mediate cellular transformation. These include six nuclear antigens and three latent membrane proteins (LMPs). In lymphoid and epithelial tumors, viral latent gene expressions have distinct pattern. In both primary and metastatic tumors, the constant expression of latent membrane protein 2A (LMP2A) at the RNA level suggests that this protein is the key player in the EBV-associated tumorigenesis. While LMP2A contributing to the malignant transformation possibly by cooperating with the aberrant host genome. This can be done in part by dysregulating signaling pathways at multiple points, notably in the cell cycle and apoptotic pathways. Recent studies also have confirmed that LMP1 and LMP2 contribute to carcinoma progression and that this may reflect the combined effects of these proteins on activation of multiple signaling pathways. This review article aims to investigate the aforementioned EBV-encoded proteins that reveal established roles in tumor formation, with a greater emphasis on the oncogenic LMPs (LMP1 and LMP2A) and their roles in dysregulating signaling pathways. It also aims to provide a quick look on the six members of the EBV nuclear antigens and their roles in dysregulating apoptosis.

## Introduction

It is currently known that viral infections are responsible for 15–20% of all human cancers (1). These oncogenic viruses have many complicated strategies that disrupt biological pathways in the infected host cells. The genetic material of these viruses undergoes several processes: replicating in harmony with the cell division of the infected host, escaping from immune surveillance, and inhibiting apoptosis (2). In addition, it increases the activities of telomerase enzyme resulting in immortality of the infected host cells (3, 4). Moreover, virus infected cells have an altered cell-to-cell adhesion properties facilitating further proliferation, transmission, and spreading of the virus particles to other areas of the body (5).

One of the best-studied example of these viruses are the herpesviruses which are prevalent in the animal kingdom. They are large double-stranded DNA viruses with a genome size of 100–200 kilobases (6). In humans, eight herpesviruses have been identified: herpes simplex virus 1 and 2 (HSV-1 and HSV-2) or human herpesvirus (HHV-1) and (HHV-2); varicella-zoster virus (VZV or HHV-3); Epstein–Barr virus (EBV or HHV-4); human cytomegalovirus (HCMV or HHV-5); human herpesviruses 6 and 7 (HHV-6 and HHV-7); and Kaposi’s sarcoma-associated herpesvirus (KSHV or HHV-8) (6).

**Figure 1:**
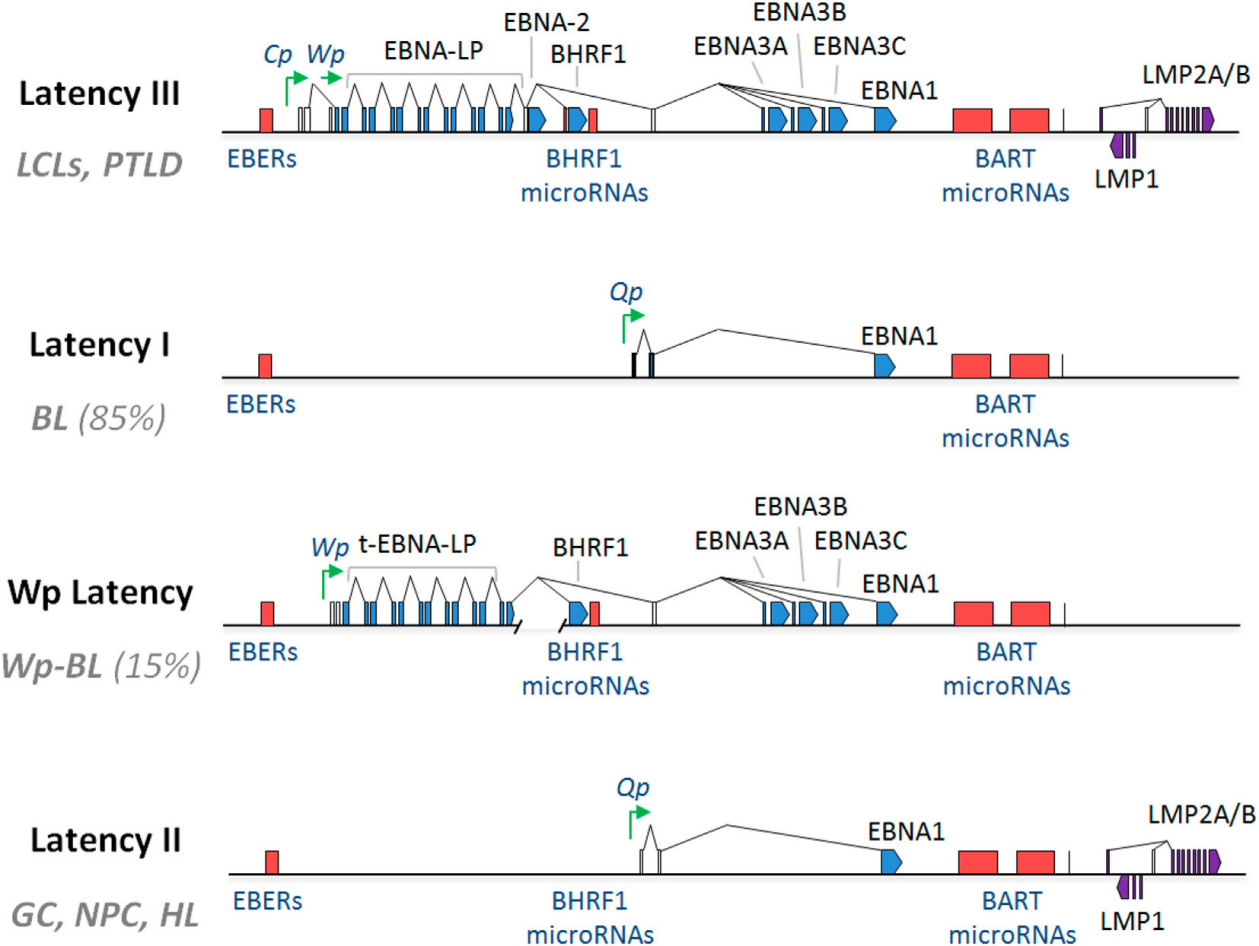
Epstein–Barr virus (EBV)-associated malignancies patterns of gene expression. Latency III EBV gene expression: found in *in vitro* transformed B cells into lymphoblastoid cell lines (LCLs); Latency I EBV gene expression: found in the majority (85%) of EBV-positive Burkitt lymphomas (BLs); Wp-restricted Latency: found in a minority (15%) of EBV-positive BLs (Wp-BL); and Latency II EBV gene expression: found in EBV-positive Hodgkin lymphoma (HL) as well as the EBV-associated epithelial malignancies, nasopharyngeal carcinoma (NPC), and gastric carcinoma (GC). Latent proteins [Epstein–Barr virus nuclear antigen (EBNA)1, EBNA2, EBNA3A, EBNA3B, EBNA3C, EBNA-LP, BHRF1, latent membrane protein (LMP)1 and LMP2A/B] are shown in blue. Non-coding RNAs [Epstein–Barr encoded RNAs (EBERs), miR-BHRF1s, and miR-BamHI A rightward transcripts (BARTs)] are shown in red, and selected latent promoters (Cp, Wp, and Qp) are shown in green. Connecting lines denote splicing patterns, while blocks indicate exons. In Wp-BL, EBNA-LP is truncated due to a genomic deletion and is therefore denoted as t-EBNA-LP (14).

Epstein–Barr virus is a HHV that causes many human B cell lymphomas, including Burkitt lymphoma (BL), Hodgkin lymphoma (HL), diffuse large B cell lymphoma, and lymphoproliferative disease in immunocompromised hosts (7, 8). Tumors infected with EBV are largely composed of latently infected cells. In this stage, the virus is still in the nuclear episome form and is replicated by the DNA polymerase of the host cell (9). EBV-positive human lymphomas have many distinctive forms of viral latency, which differ in the number of genes expressed, of which only type III EBV latency converts primary B cells into long-term lymphoblastoid cell lines (LCLs) *in vitro* (9). However, this form of latency is the most immunogenic form and usually restricted to tumors of immunosuppressed patients. Latency III represents the most extensive form of latent infection and a variety of non-coding RNAs, as well as 10 EBV-encoded proteins are expressed in this stage. These are latent membrane proteins (LMP1, LMP2A, and LMP2B), EBV nuclear antigens (EBNA-1, EBNA-2, EBNAs-3A, -3B, -3C, and EBNA-LP), and the viral BCL-2 homolog, BHRF1. In addition, two non-coding RNAs (EBER1 and EBER2) and two families of microRNAs encoded within the *BamHI* A rightward transcripts (BARTs) and the BHRF1 locus (BHRF1 miRNAs), respectively (Table 1) (10–13). These products of EBV genes are expressed at different time points after EBV infection of B cells and leading finally to growth transformation Figure 1 (14).

**Table 1:** Epstein–Barr virus (EBV) gene expression and viral latency.

Stage of EBV latency	EBV genes transcribed	Types of cells infected and tumors
Type 0	EBERs	Memory B cells

Type I	EBERs, EBNA1, BARTs	Burkitt’s lymphoma

Type II	EBERs, EBNA1, BARTs, LMP1, LMP2	Nasopharyngeal carcinoma, gastric cancer, Hodgkin’s lymphoma, NK/T lymphoma

Type III	EBERs, EBNA1, EBNA-LP, EBNA2, EBNA3A–C, BARTs, LMP1, LMP2	Lymphoblastoid cell (infectious mononucleosis), post-transplant lymphoproliferative disease, patients with immunosuppression

*BART, BamHI A rightward transcript; EBER, EBV-encoded small RNA; EBNA, EBV nuclear antigen; EBNA-LP, EBV nuclear antigen leader protein; LMP, latent membrane protein*.

Both post-transplant lymphoproliferative disorder cells and LCLs produce all six Epstein–Barr virus nuclear antigens (EBNA) and three LMPs (15). These proteins are necessary for transforming B cells, as mutated viruses that lack EBNA1, EBNA2, EBNA-LP, or LMP1 show a huge reduction in their ability to transform B cells (16–20). However, whether these proteins are sufficient for B cell transformation remains unclear. Beside these proteins, EBV genome encodes many non-coding RNAs, including the Epstein–Barr encoded RNAs (EBERs), as well as 25 miRNAs and one small nucleolar RNA (21–25). miRNAs impair the translation and reduce the stability of mRNAs—that contain complementary sequences—by direct binding to them.

**Figure 2:**
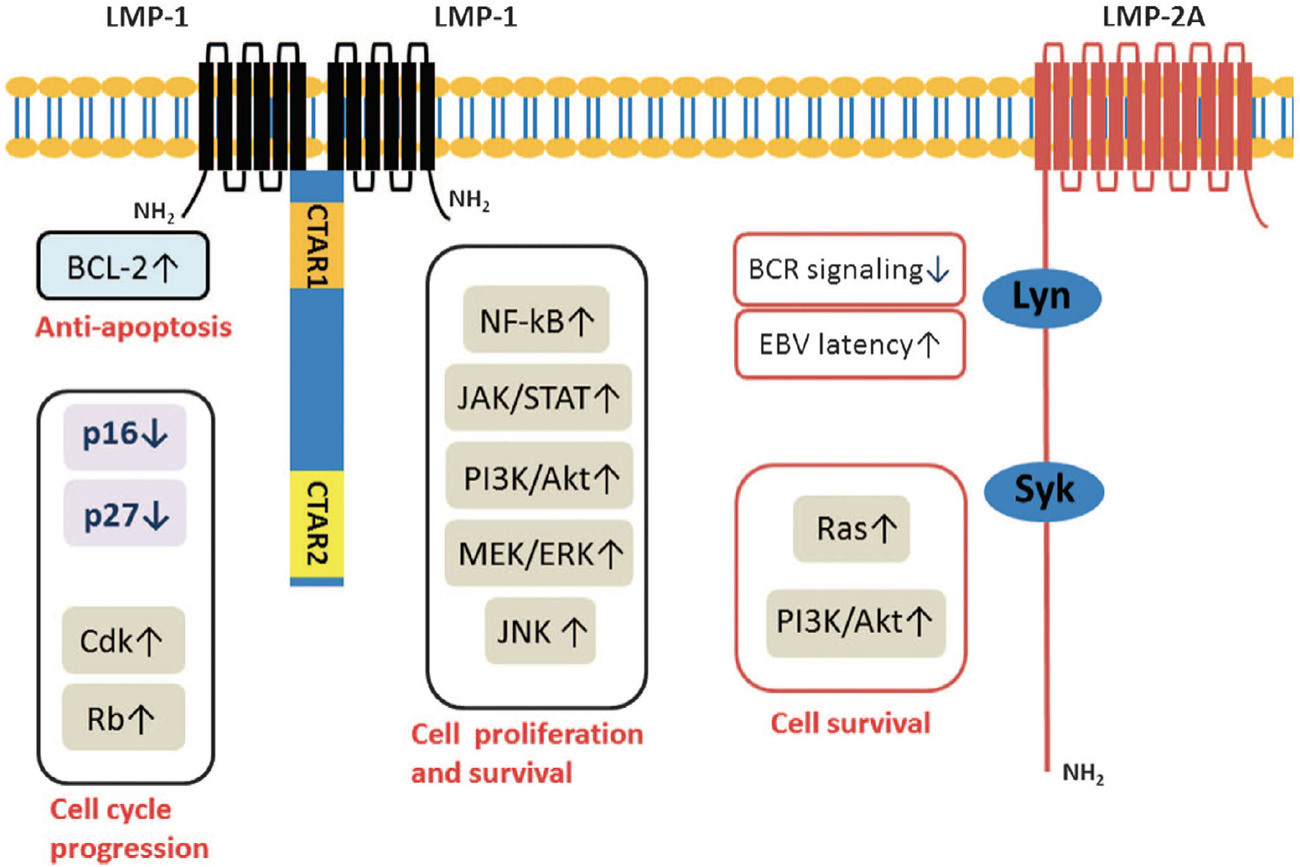
Latent membrane proteins (LMPs) (1 and 2A) downstream signal transduction pathways (28).

Recent reverse genetic analysis helped in identification that only five EBV oncoproteins and viral miRNAs are crucial for conversion of primary B-cells into continuously proliferating LCLs (26, 27). Recently it has been shown a cooperation functions between LMP1 and LMP2 toward contribution to progression of carcinomas reflected by their combined effects on activation of multiple signaling pathways (Figure 2) (28, 29).

In this review, we thought to shed lights on the EBV–LMPs (LMP1 and LMP2A) and Epstein–Barr Nuclear Antigens (EBNA-1, EBNA-2, EBNAs-3A, -3B, -3C, and EBNA-LP) due to their established roles in EBV persistence and latency. Moreover, we focused on their roles in different signal transduction pathways activation, which are critical for lymphoblastoid B-cell transformation, growth and survival, and therefore a potential therapeutic targets.

## EBV-Latent Membrane Proteins

### LMP1

The Epstein–Barr virus latent membrane protein 1 is expressed in many types of cancers, include gastric cancer, Burkitt’s lymphoma, and HL (28). It is also expressed in AIDS and post-transplant lymphomas (30). This protein has profound effects on cellular signaling pathways and growth. It modulates several processes, include migration, differentiation, and tumorigenesis (31, 32). Studies employs genetic deletion of recombinant viruses have shown that LMP1 is required as one of the LMPs for EBV-induced B-cell immortalization *in vitro* (29, 33). Significantly, LMP1 has an oncogenic function in non-lymphoid cells and it induces growth transformation in certain immortalized rodent fibroblast cells (34). *In vitro* studies have shown that heterologous expression of LMP1 lead to the loss of anchorage dependence, increased invasive capacity and inhibition of terminal differentiation in cancer cell lines (31).

Latent membrane protein 1 is an integral membrane protein with a molecular weight of 66 kDa. It consists of a short amino acid cytoplasmic N-terminus (amino acids 1–23), six transmembrane spanning regions (amino acids 24–186), and a large 200 amino acid cytoplasmic C-terminal tail (amino acid 187–386). The LMP1 transmembrane domains mediate homotypic aggregation, lipid raft association, and ligand-independent signaling from two cytoplasmic tail domains known as transformation effect site 1 (TES1) and TES2, or C-terminal activation region 1 (CTAR1) and CTAR2 (19, 35) C-terminal region contains three distinct functional domains: C-terminal activating regions 1, 2, and 3 (CTAR1, CTAR2, and CTAR3).

**Figure 3:**
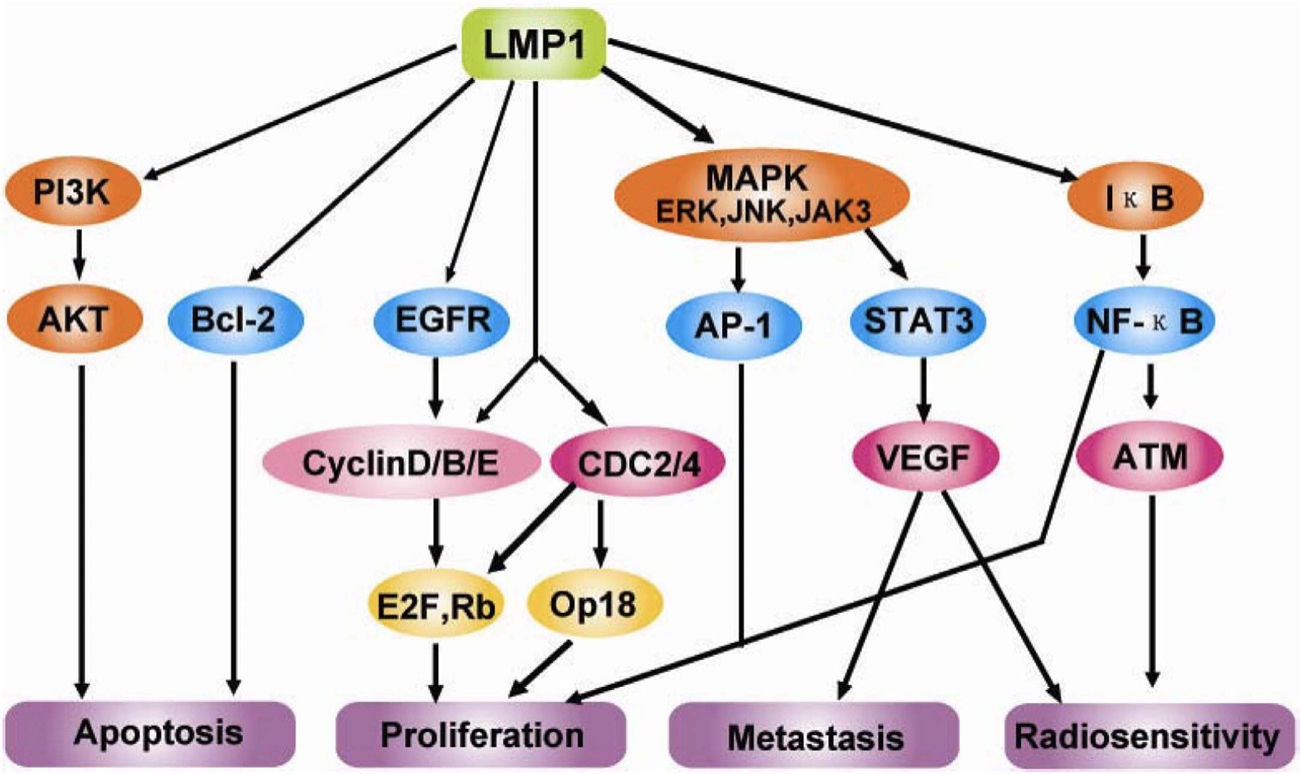
Molecular interactions and signaling pathways engaged by LMP1 in the carcinogenesis of nasopharyngeal carcinomas (NPCs). LMP1 C-terminal activation region 1 (CTAR1) regulates NIK/IKKs activation and then phosphorylates IκBα, thus activating NF-κB through TNFR-associated factor (TRAF)1, TRAF2, and TRAF3; while CTAR2 activates NF-κB through tumor necrosis factor receptor-associated death domain (TRADD) and TRAF2. Active NF-κB induces the cell immortalization *via* the upregulation of the telomerase activity through the translocation of hTERT protein bound to NF-κB, blocks the cell apoptosis *via* the upregulation of the survivin activity, and promotes the cell proliferation *via* regulating survivin, CyclinD1, CyclinE and EGFR signaling, etc. Also, LMP1 can increase the serine phosphorylation level of Annexin A2 by activating the PKC signaling pathway, which can promote the cell proliferation. LMP1 CTAR2 triggers AP-1 signaling cascade by activating ERK, P38, and the c-Jun N-terminal kinases (JNKs), members of the stress-activated group of MAP kinases, *via* the binding with TRADD/TRAF2 complex. Active AP-1 upregulates the expression of MMP9 and mediates invasion and metastasis of NPC cells. LMP1 CTAR3 between CTAR1 and CTAR2 triggers the Janus kinase (JAK3)/signal transducers and activators of transcription (STAT) signaling pathway, which can enhance VEGF transcription and expression, thereby promoting invasion and metastasis of NPC cells (42).

C-terminal activating region 1 and 2 (CTAR1 and CTAR2) are two activating regions located within the C-terminus of LMP1. CTAR1 (amino acids 186–231) is located proximal to the membrane and it is essential in primary B cells transformation by EBV. CTAR2 is located at the end of C-terminus (amino acids 351–386) and it is important for the long-term growth of EBV-positive primary B cells (36, 37). LMP1 activates many signaling pathways, include nuclear factor kB (NF-kB), c-Jun N-terminal kinase (JNK)–AP-1, p38/mitogen-activated protein kinase (MAPK), and Janus kinase (JAK)–signal transducers and activators of transcription (STAT) (Figure 3) (38–42).

**Figure 4:**
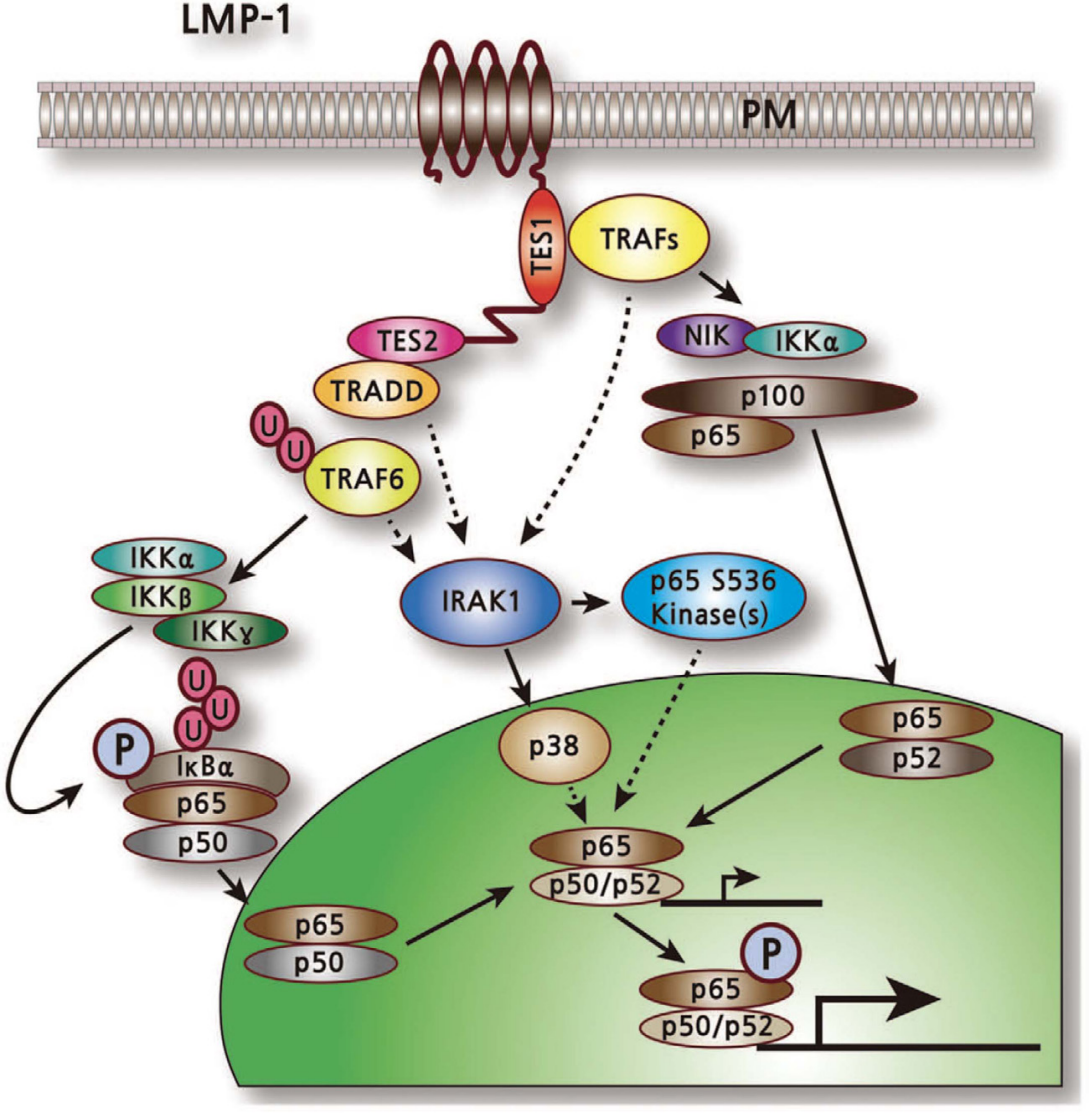
LMP1-mediated activation of nuclear factor kB pathway (43).

The first important indication of the role of LMP1 in abnormal cell signaling was the activation of the NF-kB transcription factor (Figure 4) (43). NF-kB can be activated independently by both CTAR1 and CTAR2 (38). LMP1 mutant deletion studies confirmed that CTAR2 interacts with the tumor necrosis factor receptor-associated death domain (TRADD) protein and this interaction accounts for most (70–80%) of the LMP1-mediated NF-kB activation (44). TRADD normally mediates NF-kB activation and signaling from aggregated tumor necrosis factor receptor I (TNFR-I). LMP1 interaction with TRADD is mediated by the last eight amino acids of LMP1. However, these amino acids do not define the entire activation site (44). CTAR1 define the remaining (20–30%) of NF-kB activation by LMP1, specifically the *P204xQ206xT208* motif which interacts with a number of the TNFR-associated factors (TRAFs) (45–47). The cytoplasmic tails of other TNFR members, including CD30 and CD40 are also contains the *PxQxT* TRAF binding motif.

Latent membrane protein 1 also activates JNK cascade (known as stress-activated protein kinase) (48). This pathway ends with the activation of the AP-1. LMP1 transient transfection studies suggest that CTAR2 is the only domain that induces the expression of the transcription factor AP-1 (49). Stimulation of CD40, TNFR-I, and TNFR-II with an appropriate ligand results in JNK activation which is mediated *via* a TRAF2-dependent pathway. Although both NF-kB and JNK pathways looks similar, LMP1-mediated activation of NF-kB and JNK pathways can be dissociated. Eliopoulos et al. showed that usage of a constitutively active mutated IkappaBα to inhibit the NF-kB pathway did not impair JNK signaling, whereas expression of a negative stress-enhanced kinase (c-Jun N-terminal kinase kinase) blocked the JNK signaling mediated by LMP1 but not NF-kB signaling (50).

Janus kinase 3 activation is mediated by a proline rich sequence within the 33 bp repeat of C-terminus of LMP1 and a surrounding sequence between CTAR1 and CTAR2 (40). This proline rich sequence has been tentatively referred to as CTAR3. The expression of the genes encoding LMP1 and JAK3 in 293 cells leads to enhanced JAK3 tyrosine phosphorylation and leads finally to the activation of STAT transcription (STAT1 and STAT3). LMP1-mediated activation of JAK/STAT pathway has a rapid kinetics giving rise to the fact that this pathway may precedes both NF-kB and JNK activation and might predisposes the cell to these later signals (51).

Latent membrane protein 1 also activates P38/MAPK pathway and the corresponding transcription factor ATF2. Studies employed C-terminal mutants of LMP1 have shown that CTAR1 and CTAR2 regions are important in activating p38 pathway (40). Specific inhibitors of NF-kB and P38/MAPK pathways were used to determine the relation between these two pathways. When an inhibitor of NF-kB activation was used, the activation of p38 was not impaired. Also, the use of an inhibitor of p38 did not affect the binding activity of NF-kB. These results suggested that LMP1 activates p38/MAPK and NF-kB pathways in an independent way. However, using non-functional mutant of TRAF2 to inactivate TRAF2 blocked both pathways suggesting that the two pathways diverge downstream of TRAF2 (40). LMP1 aggregation within the plasma membrane is a prerequisite for signaling irrespective of the LMP1-mediated signaling pathways. This aggregation is a transmembrane domains intrinsic property (44). LMP1 differs from TNFR family in that LMP1 serves as a constitutively activated receptor; therefore, requires no extracellular ligand binding. Chimeric molecules-based experiments using extracellular and transmembrane domains of CD2, CD4, or the nerve growth factor receptor with the cytoplasmic C-terminus of LMP1, proved that LMP1 signaling only occurred when chimera aggregation occurred *via* either ligand binding or antibody induced aggregation (44, 52). On the other hand, when the CD40 cytoplasmic tail linked to the N-terminal and transmembrane tails of LMP1, it became constitutively activated (53, 54).

Latent membrane protein 1 has the ability to transform MDCK cells by promoting an epithelial to mesenchymal transition (EMT) (54). In this cell line, the transcriptional repressor Twist is responsible for this phenomenon (55). LMP1 has been also shown to induce EMT in other epithelial cell lines, including breast (56), lung (57), and nasopharyngeal (54, 58–60). Horikawa et al. showed that overexpression of the transcriptional repressor snail is linked to LMP1 expression in NPC biopsies (55). This study showed also that EMT is induced by expression of LMP1 in a Snail-dependent mechanism. In a recent study conducted by Zuo et al. in NPC, they found that cadherin 6 is activated by LMP1 to mediate EMT and metastasis by switching from E-cadherin to K-cadherin (cadherin 6) (61). Morris et al. showed that LMP1 is able to induce EMT *via* its CTAR1 domain in MDCK cells (62). They used pharmacological inhibitors to inhibit ERK–MAPK, SFK, phosphotidylinositol 3-kinase (PI3-K), and TGFβ. They found that ERK–MAPK, SFK and PI3-K, but not TGFβ have critical roles in LMP1-mediated EMT. Ligation of β1 integrins with its cognate ligand, fibronectin was mandatory for ERK–MAPK and FAK phosphorylation by LMP1 (62).

**Figure 5:**
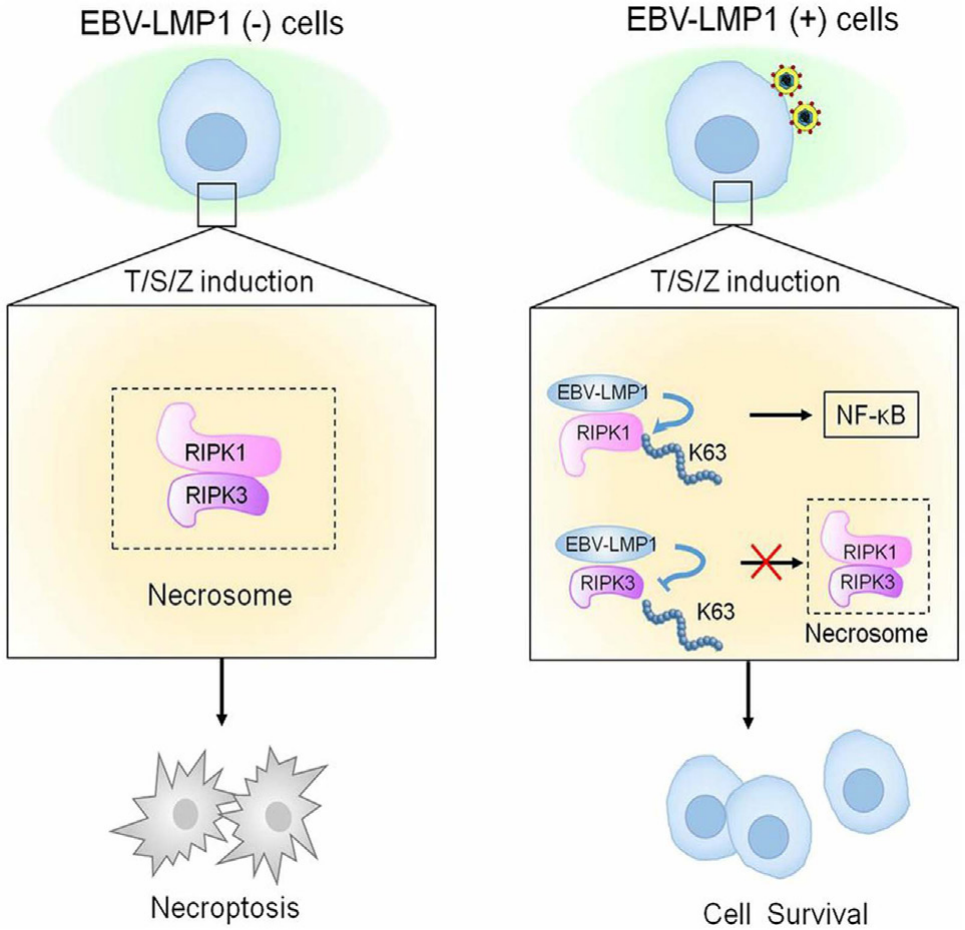
Epstein–Barr virus (EBV)–LMP1 regulates T/S/Z-induced necroptosis. EBV–LMP1 (−) cells stimulated with T/S/Z undergo necroptosis through RIPK1–RIPK3 signaling. However, EBV–LMP1 (+) cells can survive under this stimulation. On the one hand, LMP1 interacts directly with both RIPK1 and RIPK3 through its C-terminal activation region. On the one hand, LMP1 promotes K63-linked polyubiquitination of RIPK1 and suppresses the protein expression while inhibiting K63-linked polyubiquitination of RIPK3. These effects contribute to the activation of NF-κB and disruption of necrosome formation, collectively switching cell fate from death to survival (63).

In a recent study conducted by Liu et al. (63), they have showed that the γ-herpesvirus EBV blocks necroptosis in EBV-infected human nasopharyngeal epithelial cells and nasopharyngeal carcinoma (NPC) cells. In this study, LMP1 inhibit necroptosis independently from RIP homotypic interaction motif (RHIM) signaling competition as it lacks RHIM domain. CTAR of LMP1 interacts directly with both RIPK1&3. Importantly, LMP1 has the ability to modulate the post-translational modification of the two receptor-interacting proteins. In addition, LMP1 induces a switch from cell death by necroptosis to survival through promotion of K63-polyubiquitinated RIPK1, suppression of RIPK1 protein expression, and inhibition of K63-polyubiquitinated RIPK3. The authors have introduced an evidence on the ability of LMP1 to interrupt the initiation process of necroptosis before necrosome formation and hence suppression of necroptosis by EBV (Figure 5) (63).

### Latent Membrane Protein 2A (LMP2A)

**Figure 6:**
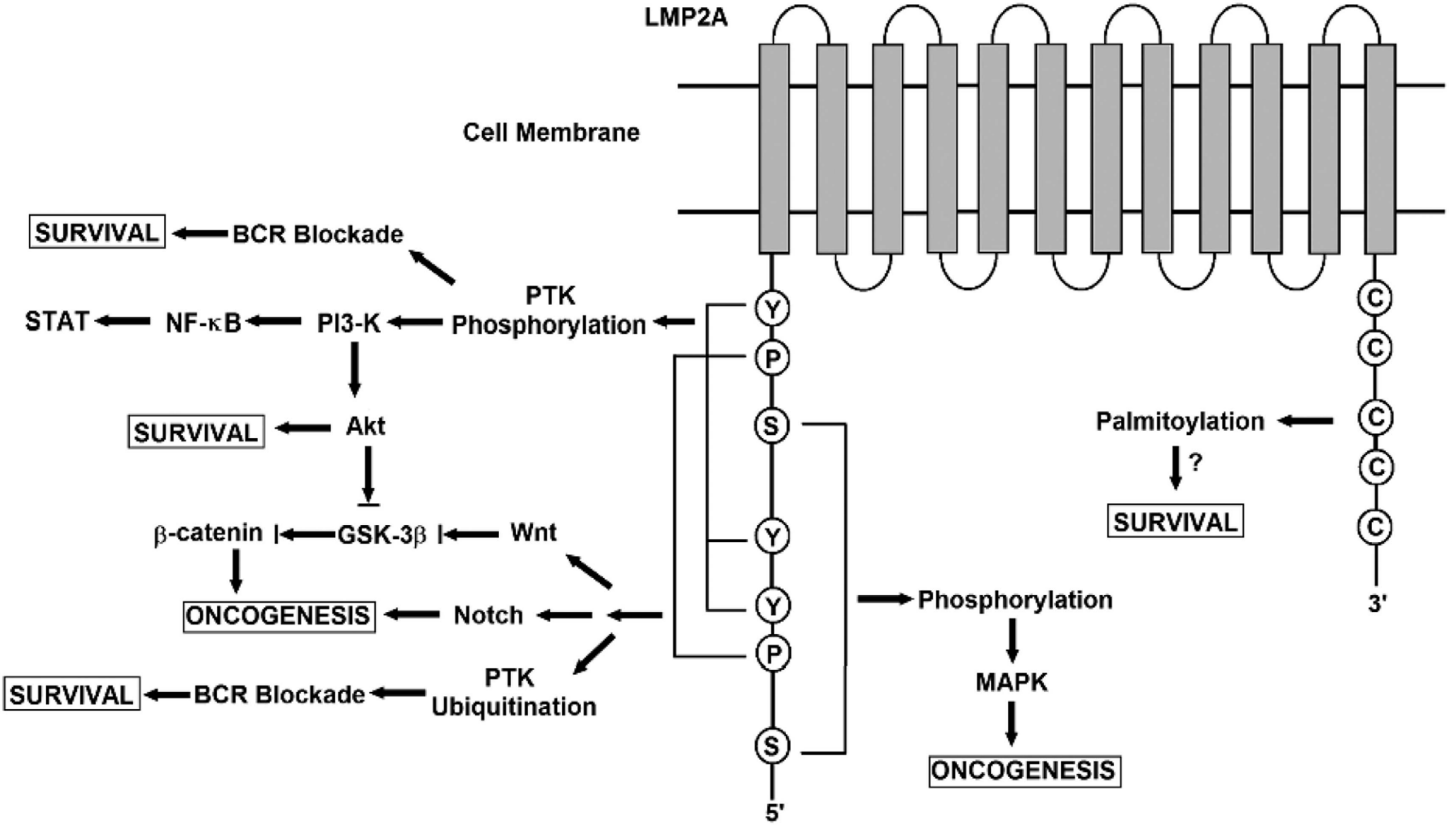
A schematic diagram showing the signaling pathways engaged by the LMP2A gene. The N terminal domain of LMP2A prevents B cell receptor (BCR) signaling by recruiting Nedd4-like ubiquitin-protein ligases and B-cell signaling molecules, leading to the degradation of LMP2A and its associated molecules in a ubiquitin-dependent manner. LMP2A also provides a survival signal to BCR-negative B-cells through the activation of the Ras/phosphotidylinositol 3-kinase (PI3-K)/Akt pathway. Activating this pathway induces the transcription of anti-apototic genes, the expression of which is controlled by NF-κ B. Notch signaling regulates various cellular processes including cell survival and proliferation. In LMP2A-expressing splenic B cells, Notch activation is reported. Notch signaling is closely related to the pathogenesis of Hodgkin lymphoma (HL). LMP2A perturbs the turnover of β-catenin and other proteins that are involved in Wnt signaling. β-catenin is stabilized and activated by LMP2A through PI3-K/Akt activation, which inhibits glycogen synthase kinase-3β (GSK-3β). The association of LMP2A with mitogen-activated protein kinase (MAPK) was implicated in the development of B-cell malignancy while the activation of MAPK was not observed in LMP2A-expressing epithelial cells. The multiple cysteine motifs within the C-terminal of LMP2A are required for LMP2A palmitoylation. Studies of the proposed palmitoylation are required for LMP2A-mediated survival signal and function as they regulate the protein interaction or localization required for LMP2A-mediated cell survival.

Latent membrane protein 2A role in malignancy remains an enigma. In NPC, LMP2A expressed at both the RNA and the protein levels (64). Also, LMP2-specific antibodies were detected in sera of NPC patients (65). Moreover, LMP2A expression is consistently detected in Hodgkin’s lymphoma and NPC tissues (66, 67). Based on these findings, LMP2A may plays specific roles in malignancy (68). Despite earlier genetic studies stated that both LMP2A and LMP2B are not essential for the transformation of B cells *in vitro* (69, 70). Another study showed that LMP2A transforming feature presents only in the immortalized epithelial cell line, but not in normal epidermal cells (71). It also presented that LMP2A expression-associated transformation properties manifests only in certain cellular contexts and generally are subtler (72). LMP2A—according to another study—is also important for growth transformation of germinal center B cells. These B cells have deleterious somatic hypermutations in their immunoglobulin genes and therefore, they do not express genuine B cell receptor (BCR). The study suggested that LMP2A has strong antiapoptotic and/or transforming features. In certain B cells, they function as an indispensable BCR mimics as in Hodgkin’s lymphoma (73). In the following sections, various signaling pathways and involvement in viral latency and malignant transformation induced by LMP2 is covered (Figure 6).

#### BCR Blockade and Ubiquitin-Mediated Pathway

Latent membrane protein 2A expression interferes with BCR signaling and function. According to previous studies, LMP2A low expression did not inhibit Ig rearrangement or BCR expression. It also did not inhibit the differentiation of normal B cells into follicular and marginal zone B cells. On the contrary, the high expression of LMP2A inhibited BCR expression and caused B-1 differentiation in bone marrow and other peripheral lymphoid organs (74–76). LMP2A negatively regulates signaling of BCR in two ways: excluding BCR from lipid rafts and targeting the Src family members of the Lyn and Syk protein tyrosine kinases marking them for degradation by ubiquitin pathway (77, 78). The BCR signal transduction blockade is achieved by either sequestering PTK away from BCR or PTK degradation by ubiquitin (79–81). In B-cell signaling, Itchy (Nedd4 ubiquitin ligase) downregulates LMP2A activity. In epithelial cells, β-catenin is activated and stabilized by LMP2A through PI3-K and Akt activation, which suppresses glycogen synthase kinase-3β (GSK-3β) (82). GSK-3β is in turn tightly regulated by Wnt signaling (83). Further studies are needed to determine the precise mechanisms by which LMP2A alters these signaling pathways during viral latency and malignant transformation.

#### MAPK Pathway

Mitogen-activated protein kinase family consists of three pathways, namely ERK/MAPK, JNK/MAPK, and p38/MAPK.

The MAPK signaling pathways are involved in different fundamental events such as proliferation, differentiation, apoptosis, and migration under normal conditions (84, 85). Abnormal regulation of these pathways leads to carcinogenesis. LMP2A activates MAPK signaling in various EBV-infected cell lines according to several evidences *in vitro* (86–88). For example, one study on lymphoblastoid B-cell lines and BL cell lines suggested that LMP2A activates ERK/MAPK (88). Another study employed LMP2A transgenic mice stated that the continuous activation of ERK/MAPK and PI3-K/Akt pathways leads to proliferation and survival (89). c-Jun is a crucial downstream effector of the JNK/MAPK pathway. It is induced as an early factor under mitogen stimulation and it is playing a crucial rule in cell growth (90, 91). Moreover, it is a potent inhibitor of differentiation. In organic raft cultures, LMP2A is able to transform and inhibit keratinocytes differentiation (92–94). These observations link LMP2A to JNK/MAPK. According to microarray studies, alterations of gene transcription of several MAPK-related molecules are induced by LMP2A, including upregulation of Ras, its homolog Ras GTPase-activating protein-binding protein 1, MAPK2K1, MAPK2K2, and the suppression of Ras suppressor protein (88). Activated Ras cooperates with c-Jun for effective transformation (95, 96). The exact mechanism and molecular interaction of LMP2A in MAPK signaling remains unclear. In addition, our picture of the involvement of LMP2A in MAPK signaling derived from *in vitro* studies on different cell lines but there are no sufficient *in vivo* studies to support the connection between LMP2A and MAPK signaling.

#### The PI3-K/AKT Pathway

Phosphotidylinositol 3-kinase/Akt signaling pathway has an important role in transformation, antiapoptotic properties, adhesion, and invasion (97–101). Numerous studies have suggested that LMP2A activates PI3-K/Akt signaling which leads to cell growth enhancements and antiapoptotic effects in B-cells, lymphoma, gastric carcinoma (GC), and epithelial cells (73, 102, 103). When LY294002 is used as an inhibitor of PI3-K, it resulted in the inhibition of colony formation induced by LMP2A in soft agar. This phenomenon indicates that activation of PI3-K is critical for anchorage-independent growth of epithelial cells (104, 105). Treatment of B-cells derived from LMP2A transgenic mice with specific inhibitors of Ras, PI3-K, and Akt made these cells very sensitive to apoptosis. These results suggest that LMP2A activates Ras followed by the PI3-K/Akt pathways, ending in B-cell survival (104). In cell lines of human Burkett’s lymphoma and GC, usage of PI-3K inhibitors blocked the LMP2A-dependent apoptotic effects. This demonstrates that the LMP2A anti apoptotic effects depend on the PI3-k signaling pathway (105).

TGF-β1 induces apoptosis by activating caspases (106–109). TGF-β1-induced caspase activity and apoptosis is inhibited by LMP2A through activation of PI3-K/Akt pathway *via* Akt phosphorylation at its serine residue (105, 110). This is supported by increased level of activated Ras and Bcl-XL expression resulted in suppression of B-cell apoptosis (73). Integrin is the other pathway regulated by LMP2A-associated PI3-K/Akt. Integrin-dependent PI3-K activation leads to invasive and adhesive phenotypes, and therefore the protection of apoptosis (101, 111). PI3-K/Akt pathway activation plays an important role in EBV-associated malignancies. It maintains EBV persistence and latency but not cell transformation (102, 103). The aggressive tumorigenicity of epithelial cells is maintained due to the activation of Ras/PI3-K/Akt by LMP2A, alongside other genetic changes (103). The regulation of this pathway and its differential expression in different types of EBV-induced tumors have also yet to be discovered.

#### The NF-κB and STAT Pathway

Signal transducers and activators of transcription and NK-κB pathways constitutive activation occur in malignancies, commonly due to genetic or autocrine/paracrine alteration (74, 112). NF-kB activation in epithelial cells induces production of IL-6 and activates STAT (74, 113) which results in cell growth and survival. Moreover, it mediates inflammatory responses through induction of cytokines and chemokines production. This results in the stimulation of anti-tumor activity *via* the recruitment and activation of immune cells (113). Altering the balance between the tumorigenesis and the anti-tumor immune response in NF-kB pathway results in tumor development (74).

In human carcinoma cell lines infected by EBV, LMP2A downregulates the STAT and NF-kB pathways *in vitro*. This fact was tested by using wild-type (wt) recombinant EBV (rEBV) and mutant rEBV, in which the LMP2A gene is deleted (rEBV-2A) (114). The results showed that the transient expression of LMP2A in LMP2A-deficient carcinoma cells suppressed LMP1 expression, IL-6 secretion, STAT, and NF-kB activities. On the contrary, the downregulation of LMP2A resulted in the induction of LMP1 (114).

Nuclear factor kB pathway regulates the production of IL-6 (113). In rEBV HONE-1 cells, transfection of a recombinant adenovirus expressing mutant IkBα and the luciferase reporter showed that IL-6 promoter activity was noticeably decreased (114). These results suggested that LMP2A has an important role in modulating STAT pathways and in modulating LMP1 expression indirectly through NF-kB activity in epithelial cells (114). Both STAT and NF-kB contributes to various cancer phenotypes in EBV-associated malignancies. For example, NF-kB suppression induces epidermal hyperplasia which ends in developing the undifferentiated tumor; NPC (115). Akt also positively regulates NF-kB which leads to an increased level of Bcl-xL in B-cells, ensuring an antiapoptotic effect and cell survival (116).

## EBV Nuclear Antigens

### Epstein–Barr Nuclear Antigen 1

Epstein–Barr nuclear antigen 1 is essential for viral DNA replication and episome maintenance during cellular replication at latent stages in infected cells. Besides, it is the only protein that expressed in all EBV-associated tumors (105, 117–120). It has no enzymatic activity and it is not clear how it initiates and maintains EBV genome (117–119). It has been reported that EBNA1 is associated with the survival of Burkitt’s lymphoma cells and response to DNA damage in NPC. A possible mechanism is modulating of ROS content through regulation of nicotinamide adenine dinucleotide phosphate oxidase enzymes (121–123). Additional studies have reported that EBNA1 contribute to gastric cancer development through loss of promyelocytic leukemia nuclear bodies (123, 124).

DNA replication and episome maintenance functions of EBNA1 are due to its ability to bind to certain elements of DNA within the EBV origin of plasmid replication (OriP). EBNA1 requires the family of repeats (FR), which composed of 20 tandems 30 bp repeats to make the metaphase chromosome tethering and transcriptional enhancer activities (117, 118, 124, 125). Away ~1 kb from the FR is located the dyad of symmetry, which composed of four EBNA1 binding sites and enables EBNA1 to initiate DNA replication (117, 118, 124, 125). EBNA1 is able to interact with both elements simultaneously through a DNA looping mechanism (126–128). The binding of EBNA1 to OriP is critical for replication and maintenance of episome. EBNA-1 reportedly binds and regulates the promoters of many other cellular genes but the functional consequences and implications of these interactions for cell survival are not yet fully elucidated (128–132).

### EBNA-2 and EBNA-LP

Following EBV infection of B cells, EBNA-2 and EBNA-LP are the first proteins to be expressed. EBNA2 expression is essential for B cell transformation (133). EBNA2 is a functional mimic of cellular Notch (133–135). Also, it has RBP-Jκ-mediated pleiotropic effects on chromatin organization and gene regulation which makes EBNA2 responsible for starting cell cycle (136–139). EBNA2 can directly bind and inhibit Nur77 (140, 141). Nur77 is an orphan nuclear receptor that binds and modulates the functions of several pro-survival BCL-2 family members (142). Moreover, expression of EBNA2 was shown to decrease the expression of BIK, the BCL-2 family death inducer (143). It was also shown that EBNA2 expression upregulates BFL-1/A1 (pro-survival BCL-2 family protein) at mRNA level *via* binding to RBP-Jκ/CBF1 (144). Recently, EBNA2 also contributes to MYC activation through long-range interaction (145). MYC has an opposing function as it can both increase proliferation and sensitize cells to apoptosis (146). Another nuclear antigen essential in B cell transformation is EBNA-LP (18, 147–149). It acts as a transcriptional coactivator of EBNA2 (16) and has few survival functions attributed to it in the context of LCLs. It has been reported that EBNA-LP can bind to Fte-1/S3a, which contributes to cell survival by interacting with PARP (150). However, another study found that in a yeast 2-hybrid system, EBNA-LP could interact with p14^ARF^ and colocalized with p14^ARF^ and p53 transcripts in LCLs (151). Additionally, in COS-7 (the primate kidney cell line) EBNA-LP has also been reported to interact with BCL-2 in the presence of HAX-1 in pull down experiments using glutathione S-transferase fusion proteins (151). Therefore, EBNA-LP seems to have survival functions in transformation which merit further investigations.

### EBNA-3A, -3B, and -3C

The EBNA-3s (3A, 3B, and 3C) are a family of three large proteins, which function as regulators of virus and host cell transcription. They likely arose by gene duplication. These proteins, like EBNA-2 don’t bind DNA directly, but interact with transcription factors, such as RBP-Jk (for which all four EBNAs compete) to transactivate or repress gene expression (152). They show structural similarity; despite they share less than 30% amino acid composition (153, 154). In addition, they overlap in some of the loci and processes they regulate. Some studies have shown that only EBNA-3C is essential for B cell transformation, although when B cells infected with EBNA-3A-lacking viruses, they displayed growth impairment and quickly undergo apoptosis (155–157). On the contrary, EBNA-3B is essential for the transformation of B cells. In one study, LCLs generated with an EBNA-3B knockout (KO) virus showed high resistance to apoptosis compared to those produced with wt EBV (158, 159). Analysis of cells infected with EBNA-3 KO or estrogen-inducible EBNA-3 proteins conditional viruses showed that EBNA-3A and -3C cooperate to downregulate-through epigenetic silencing- the tumor suppressors p16INK4a and p14ARF (155, 156, 160–163) as well as downregulate the apoptosis inducing, BH3-only protein BIM. Moreover, EBNA-3C can interact with p53 as well as binding and stabilizing its regulators, ING4, ING5, MDM2, and Gemin3 (164–167). The EBNA3 proteins have the ability to regulate many genes up to 50 kb away from transcriptional start sites (TSS) (164, 168) despite EBNA-3A and -3C downregulate BIM and p14ARF at TSS through epigenetic silencing (156, 160–163, 169, 170). The EBNA-3 proteins have been estimated to collectively bind to more than 7,000 sites on the cellular genome. Therefore, extensive studies are needed to unravel many other cell survival genes regulated by the EBNA3s.

## Conclusion

Further studies both *in vivo* and *in vitro* are required to elucidate the molecular crosstalk between EBV transformed tumor cells and the tumor microenvironment. These studies are essential to define the precise mechanisms in EBV-induced oncogenesis, and to enable further insights into EBV-associated malignancies. Moreover, more studies are needed to unravel the roles of these oncoproteins in dysregulating other forms of cell death like necroptosis which could serves as a potential alternative strategy of programmed cell death to apoptosis, hence a possible therapeutic target.

## Author Contributions

AM designed the topics and contributed in writing. AE-S designed the topics and wrote the entire manuscript. LZ contributed in writing and reviewed the manuscript.

## Conflict of Interest Statement

The authors declare that the research was conducted in the absence of any commercial or financial relationships that could be construed as a potential conflict of interest.
